# Prevalence of inherited metabolic disorders among newborns in Zhuzhou, a southern city in China

**DOI:** 10.3389/fgene.2024.1197151

**Published:** 2024-02-06

**Authors:** Hunjin Luo, Jiqing Wang, Junfeng Chen, Huijian Yi, Xiaodong Yang, Yao Peng, Liu Ni, Yi-Qiong Yang, Xiao-Min Zhang, Hongping Huang

**Affiliations:** Women and Children Healthcare Hospital of Zhuzhou, Zhuzhou, Hunan, China

**Keywords:** inherited metabolic disorders, neonates, metabolites, tandem mass spectrometry, sanger sequencing, next-generation sequencing

## Abstract

**Background and aims:** Defective enzymes, cofactors, or transporters of metabolic pathways cause inherited metabolic disorders (IMDs), a group of genetic disorders. Several IMDs have serious consequences for the affected neonates. Newborn screening for IMDs is conducted by measuring specific metabolites between 3 and 7 days of life. Herein, we analyzed the incidence, spectrum, and genetic characteristics of IMDs in newborns in the Zhuzhou area.

**Methods:** Tandem mass spectrometry was conducted on 90,829 newborns who were admitted to the Women and Children Healthcare Hospital of Zhuzhou and requested for screening for IMDs. These newborns were subsequently subjected to next-generation sequencing and further validated using Sanger sequencing.

**Results:** 30 IMDs cases were found in 90,829 cases of newborns screened for IMDs, and the overall incidence was 1/3,027. The incidence of amino acid, organic acid, fatty acid oxidation and urea cycle disorders were 1/8,257, 1/18,165, 1/7,569, and 1/45,414, respectively. Additionally, 9 cases of maternal IMDs were found in our study, and unreported gene mutations of 3 cases IMDs were identified.

**Conclusion:** Our data indicated that IMDs are never uncommon in zhuzhou, meanwhile, we also found that primary carnitine deficiency was the only disorder of fatty acid oxidation in Zhuzhou, and the incidence (1/7,569) was higher than the national level, organic acid metabolic diseases are mostly inherited. Therefore, our study has clarified the disease spectrum and genetic backgrounds, contributing to the treatment and prenatal genetic counseling of these disorders in this region.

## 1 Introduction

Defects in enzymes, cofactors, or transporters of metabolic pathways cause inherited metabolic disorders (IMDs), a group of genetic disorders (Scala et al., 2012; Scaturro et al., 2013). Several IMDs have serious consequences in affected neonates, including drowsiness, irreversible intellectual disability, physical disability, coma, and even death (Saudubray et al., 2002; Wasant et al., 2002). The genealogy and incidence of genetic metabolic diseases vary greatly among different regions and ethnic groups (Chace et al., 2003). Mass spectrometry is characterized by high throughput, efficiency, and sensitivity, and can simultaneously detect several metabolites (Millington et al., 1990; Rinaldo et al., 2006; Fernandez-Lainez et al., 2012). It is widely used in screening neonatal genetic metabolic diseases and is crucial in the early diagnosis of amino acid metabolic (Wilcken et al., 2003; Shi et al., 2012), organic acid metabolic, and fatty acid oxidation diseases. Tandem mass spectrometry (MS/MS)-based NBS was launched in 2004 in China, and the average incidence of IMDs in a preliminary study was 1/3,795 (Shi et al., 2012). In recent years, proteomics has been applied to the study of IMDs, and tandem mass spectrometry (MS/MS) technology has made key advances achieved in this field. This technology enables the analysis of large numbers of proteins in different body fluids (serum, plasma, urine, saliva, tears) with a single analysis of each sample, and can even be applied to dried samples (Chantada-Vazquez et al., 2022). Meanwhile, with the introduction of next-generation sequencing (NGS) for newborn screening (NBS), more inherited metabolic disorders (IMDs) can technically be included (Veldman et al., 2023). In this study, we retrospectively analyzed the screening results of various genetic metabolic diseases of 90,829 neonates born in Women and Children Healthcare Hospital of Zhuzhou from 2019 to 2022 based on 4 years of experience in MS/MS to understand the disease spectrum, incidence, and clinical phenotype characteristics of amino acid metabolic, organic acid metabolic, and fatty acid oxidation diseases.

## 2 Materials and methods

### 2.1 Newborns

From 2019 to 2022, 90,829 newborns born in the Women and Children Healthcare Hospital of Zhuzhou were screened for various genetic metabolic diseases using MS/MS. Written informed consent was obtained from all the guardians of the infants. This study was approved by the Women and Children Healthcare Hospital of Zhuzhou (ZZFY2022IRB06).

### 2.2 Reagents and instruments

The NeoBase™ nonderivatized MS/MS kit is a product of the PerkinElmer Company, and the series mass spectrometry analysis system (ACQUITY TQD) is a product of the Waters Company. The cutoff values were initially set with reference to the worldwide collaborative project and other screening centers (Niu et al., 2010), and as the number of samples increased, they were adjusted over time.

### 2.3 Sample collection, receiving, and MS/MS

Blood samples were collected using a heelstick and spotted on Whatman 903 filter paper. Blood collection for NBS is recommended between 3 and 7 days of life. Dried blood spot (DBS) samples were delivered using cold-chain transportation to the Women and Children Healthcare Hospital of Zhuzhou within 5 days. The DBSs with a diameter of 3.2 mm were collected using an automatic drilling instrument, and the amino acids, free carnitines, and 30 acylcarnitines in the DBSs were analyzed using the NeoBase™ nonderivative kit and MS/MS system (Waters-ACQUITY TQD). The internal standards of 12 amino acids and 8 acylcarnitines were used. The cut-off values were initially set with reference to the worldwide collaborative project and other screening centers. Meanwhile, the screening indicators and their positive cut-off values were set according to the disease characteristics and were adjusted over time as the number of samples increased. Furthermore, retrospective revisions were annually made. Newborns with the results of clear aberrant initial screening were immediately referred for confirmatory tests, including biochemical and genetic analyses, and newborns with the results of mild initial screening were recalled for repeated tests. If the second test was still positive, the patient was referred for confirmatory tests.

### 2.4 Genetic analysis

Genetic analysis was performed in the Genuine Diagnostics Company (Guangzhou, Guangdong, China). Briefly, the Sanger–Cusson method was used to sequence the exons and adjacent introns for positive cases of a certain disease. DBSs were used for whole-exome high-throughput sequencing for cases of multiple diseases corresponding to the same screening indicators, and the Sanger–Cusson method was used to verify the positive sites. The PubMed database, Human Gene Mutation Database (HGMD), ClinVar, OMIM database, and the 2020 edition of ACMG guidelines were searched to identify the pathogenic mutations.

### 2.5 Diagnosis

Metabolic disease specialists made the definitive diagnoses based on the patients’ biochemical performance, genetic mutations, and clinical symptoms. Only patients diagnosed using genetic analysis were included in this study.

### 2.6 Statistical analysis

For cost calculation, the disease incidence rates were obtained from the NBS Management Information System, which covered all medical procedures for each NBS participant. Eleven diseases were diagnosed in 90,829 newborns who underwent MS/MS screening.

## 3 Results

### 3.1 Screening results of IMDs in 90,829 newborns

In total, 90,829 newborns were included in this study. A total of 1,089 suspected positive cases were screened using MS/MS, and the initial screening positivity rate was 1.199%. A total of 1,067 cases (97.9%) were successfully recalled, and the biochemical and gene sequencing results were combined to finally diagnose 30 IMD cases (Table 1), with a positive predictive value of 2.7%. Eleven IMD types were detected in 30 confirmed cases, with 1/3,027 as the overall IMD detection rate. Eleven, five, and twelve patients with amino acid (36.6%), organic acid (16.6%), and fatty acid oxidation (40%) disorders, respectively, were found. The incidences of amino acid metabolism, organic acid metabolism, and fatty acid oxidation metabolism disorders were 1/8,257, 1/18,165, and 1/7,569, respectively. Additionally, nine patients with maternal diseases were identified using our MS/MS NBS system (Table 2).

**TABLE 1:** Positive cases detected by MS/MS newborn screening.

Disorders	Positive cases	Frequency
Amino acid disorders	11(36.6%)	1/8,257
Phenylalanine hydroxylase deficiency (PAHD)	5	
Citrin deficiency (CD)	4	
Homocysteinemia (HCY)	1	
Hypermethioninemia (MET)	1	
Organic acid disorders	5(16.6%)	1/18,165
Short/branched chain acyl-CoA dehydrogenase deficiency (SCADD)	1	
Methylmalonic academia (MMA)	1	
Middle/branched chain acyl-CoA dehydrogenase deficiency (MCADD)	1	
Holocarboxylase synthetase deficiency (HCSD)	1	
Glutaric acidemia type I (GA-I)	1	
Fatty acid oxidation disorders	12(40%)	1/7,569
Primary carnitine deficiency (PCD)	12	
Urea cycle disorders	2(6.6%)	1/45,414
Ornithine transcarbamylase deficiency (OTCD)	2	
Total numbers	30	1/3,027

**TABLE 2:** Biochemical and genetic characteristics of nine patients with maternal disorders.

No.	Disorders	Abnormal parameter and concentration (μmol/L)	Affected gene	Genotype	
1	Primary carnitine deficiency (PCD)	C0: 3.56	SLC22A5	c.1400C>G (p.S467C)	c.1400C>G (p.S467C)
2	Primary carnitine deficiency (PCD)	C0: 4.37	SLC22A5	c.760C>T (p.R254*)	c.1400C>G (p.S467C)
3	Phenylalanine hydroxylase deficiency (PAHD)	Phe: 767.32 Phe/Tyr: 12.23	PTS	c.259C>T (p.P87S)	c.259C>T (p.P87S)
4	Phenylalanine hydroxylase deficiency (PAHD)	Phe: 428.95; Phe/Tyr:8.03	PAH	c.728G>A (p.R243Q)	c.728G>A (p.R243Q)
5	Phenylalanine hydroxylase deficiency (PAHD)	Phe: 311.25; Phe/Tyr: 6.23	PAH	c.728G>A (p.R243Q)	c.1174T>A (p.F392I)
6	Homocysteinemia (HCY)	Met:269.39	CBS	c.377T>C (p.M126T)	c.526G>A (p.E176K)
7	Hypermethioninemia (MET)	Met:217	MAT1A	c.433G>A (p.E145K)	c.529C>T (p.R177W)
8	Glutaric acidemia type I (GA-I)	C5DC + C6OH: 5.58	GCDH	c.416C>T (p.S139L)	c.892G>A (p.A298T)
9	Holocarboxylase synthetase deficiency (HCSD)	C5OH + C4DC: 1.56	HLCS	c.1522C>T (p.R508W)	c.1544G>A(p.S515)

### 3.2 Results of amino acid disorders

Four types of amino acid metabolic disorders (Phenylalanine hydroxylase deficiency (PAHD), citrin deficiency (CD), Hypermethioninemia (MET), and homocysteinemia (HCY) were detected, and PAHD deficiency was the most common (45.4%), followed by citrin deficiency (36.3%). Only one MET case (9%) and one HCY case (9%) were found. Among these cases, the phenylalanine concentration and phenylalanine/tyrosine ratio in patients with BH4D were significantly higher than those in patients with PAHD. Methionine concentrations in patients with MET and HCY were 217 and 269.39 μmol/L, respectively, which were higher than the reference range (reference range: 6.5–38 μmol/L) (Table3). The main mutations in the *PAH* gene of PAHD were c.728G>A (p.R243Q), c. 1,174> (p.F392I), c.259C>T (p.P87S), c.728G > A (p.R243Q), and c.433G > A c. C (p.S2R), and the mutations in the *CBS* gene of HCY were c.377T>C (p.M126T) and c.526G>A; (E176k). The mutations in the *MAT1A* gene of MET were c.433G>A (p.E145K) and c.529C>T; R177W). Four patients with CD had four different mutations in the *SLC25A13* gene.

**TABLE 3:** Levels of abnormal parameters for different disorders of amino acid metabolism.

Amino acidemias (n = 11)	n (%)	Abnormal parameter	Concentration mean (μmol/L)	References range (μmol/L)
Phenylalanine hydroxylase deficiency (PAHD)	5 (45.4)	Phe	311.25**/**323.45**/**328.23**/**348.65**/**767.32	23–95
		Phe/Tyr	6.23**/**6.78**/**6.98**/**8.03**/**12.23	0.2–1.4
Citrin deficiency (CD)	4 (36.3)	Cit	41.19/56.53/57.32/93.53/	5.5–28
		Cit/Arg	3.23/4.44/4.45/9.56	0.35–15
Homocysteinemia (HCY)	1 (9)	Met	269.39	6.5–38
		Met/Phe	8.72	0.1–0.7
hypermethioninemia (MET)	1 (9)	Met	217	6.5–38
		Met/Phe	3.48	0.1–0.7

### 3.3 Organic acid disorders and no reported gene mutations

Five types of organic acid disorders were detected in Zhuzhou: short-chain acyl-CoA dehydrogenase deficiency (SCADD), methylmalonic cademia (MMA), middle/branched chain acyl-CoA dehydrogenase deficiency (MCADD), holocarboxylase synthetase deficiency (HCSD), and type I glutaric cademia (GA-I) (Table 4). The incidence rate was 1/18,165, which was relatively low. Preliminary NBS results showed that C4 concentration with SCADD was slightly increased; the C4/C2 and C4/C3 ratios were higher than the normal range; C6, C8, and C6DC concentrations with MCADD was increased; the C5 concentration of MMA was high; and the C3/C0 and C3/C2 ratios were beyond the normal range (Table 4). Ten different mutations in five genes (*GCDH*, *ACADM*, *ACADS*, *HLCS*, and *MMACHC*) were identified in the five patients with organic acid disorders. Notably, the three mutations (c.893G>A (p.R298K), c.482G>A (p.S161N), and c.1544G>A (p.S515)) of three of the genes (*ACADM*, *ACADS*, and *HLCS*) were not reported (Table 5). Additionally, mutations in two (*ACADM* and *ACADS*) of the five organic acid disorders were paternally inherited, whereas mutations in the other two diseases (HLCS and GA-I) were maternally inherited.

**TABLE 4:** Levels of abnormal parameters for different organic acid disorders.

Organic acidemias (n = 5)	n (%)	Abnormal parameter	Concentration mean (μmol/L)	References range (μmol/L)
Short/branched chain acyl-CoA dehydrogenase deficiency (SCADD)	1 (20)	C4	1.34	0.03–0.48
		C4/C2	0.06	0.004–0.04
		C4/C3	0.77	0.04–0.4
Methylmalonic academia (MMA)	1 (20)	C3	7.44	0.35–3.8
		C3/C0	0.41	0.01–0.2
		C3/C2	0.62	0.03–0.2
Middle/branched chain acyl-CoA dehydrogenase deficiency (MCADD)	1 (20)	C6	0.47	0.03–0.35
		C8	10.88	0.02–0.15
		C6DC	0.95	0.03–0.25
Holocarboxylase synthetase deficiency (HCSD)	1 (20)	C5OH + C4DC	1.56	0.07–0.37
Glutaric acidemia type I (GA-I)	1 (20)	C5DC + C6OH	5.58	0.03–0.24

**TABLE 5:** Identification three novels mutation in disorders of amino acid metabolism and organic acid disorders.

Disorders	Affected gene	Genotype	Reported
Short/branched chain acyl-CoA dehydrogenasedeficiency (SCADD)	ACADM	c.893G>A(p.R298K)	No reported
		c.1097G>A (p.G366E)	reported
Middle/branched chain acyl-CoA dehydrogenase deficiency (MCADD)	ACADS	c.482G>A(p.S161N)	No reported
		c.1031A>G (p.E344G)	reported
Holocarboxylase synthetase deficiency (HCSD)	HLCS	c.1544G>A(p.S515)	No reported
		c.1522C>T (p.R508W)	reported

### 3.4 Fatty acid oxidation disorders (FAODs) and urea cycle disorder

Only one type of fatty acid metabolic disease–primary carnitine deficiency (PCD)–was diagnosed in Zhuzhou. Twelve patients were diagnosed with a 1/7,569 incidence rate. All infants diagnosed with PCD had low free carnitine levels, with an average C0 of approximately 4.72 (Table 6). Mutations in the *SLC22A5* gene were identified in 12 cases of neonatal PCD. The most common mutation was c.1400C>T (p.S467C), followed by c.760C>T (p.R254X), c.51C>G (p.F17L), and c.797C>T (p.P266L). Additionally, two cases of urea cycle disorders (ornithine transcarbamylase deficiency) were detected. Both patients had low citrulline levels and their Orn/Cit ratios were higher than the normal range (Table 6).

**TABLE 6:** Levels of abnormal parameters for different disorders of fatty acid oxidation disorders and Urea cycle disorders.

Fatty acid oxidation disorders and urea cycle disorders (n = 14)	n (%)	Abnormal parameter	Concentration mean (range) (μmol/L)	References range (μmol/L)
Primary carnitine deficiency (PCD)	12 (85.7)	C0	4.72 (3.56–7.94)	9–50
Ornithine transcarbamylase deficiency (OTCD)	2 (14.3)	Cit	1.4**/**1.6	5.5–28
		Orn/Cit	43.94**/**50.21	2.5–20

## 4 Discussion

MS/MS has become the main means to expand the screening of newborn IMDs. It can screen 40 to 50 disease types, including amino acid metabolism, organic acid metabolism, and fatty acid oxidation disorders (Schulze et al., 1999; Lindner et al., 2011; Ombrone et al., 2016). Newborn screening is an effective disease prevention measure, which can prevent children’s intellectual disability and improve the quality of the birth population (Chace et al., 1999; American College of Medical Genetics Newborn Screening Expert, 2006). The data used in this study were obtained from the counties and districts of the city, covering the city’s urban and suburban towns. From 2019 to 2022, 90,829 newborns were screened for 48 IMDs using MS/MS, and 11 diseases of four types (amino acid metabolic, organic acid, fatty acid oxidation, and urea cycle disorders) were detected. The total incidence rate was approximately 1/3,027. The incidence rates of neonatal amino acid metabolic, organic acid metabolic, and fatty acid oxidation diseases were approximately 1/11,831, 1/16,007, and 1/6,977, respectively. China is a large population, and according to the overall incidence of neonatal IMDs published by the state, about 3,000 patients accumulate every year. If these children are diagnosed and treated promptly, intellectual disability can be avoided. This has significant social and economic benefits.

The incidence rate of amino acid disorders in our study was lower (1/8,257) than that in Germany (26.3/100,000), the United Kingdom (26.7/100,000), and China (17.1/100,000) (Sanderson et al., 2006; Huang et al., 2022) and was even lower than that in neighboring Guangzhou (1/11,831) (Tang et al., 2021). Four amino acid disorders types were detected in our study, and PAHD accounted for the highest proportion of amino acid metabolic diseases. Some were mild cases, indicating that the incidence rate of amino acid metabolic diseases in this region is relatively low. In the future, primary prevention is expected to reduce such diseases to a lower level to improve the quality of the birth population.

Five types of organic acid metabolic diseases (short/branched chain acyl-CoA dehydrogenase deficiency (SCADD), methylmalonic cademia (MMA), middle/branched chain acyl-CoA dehydrogenase deficiency (MCADD), holocarboxylase synthetase (HCSD), and glutaric cademia type I (GA-I) were detected. The incidence of organic acid metabolic diseases in our study was 1/18,165, which was lower than the national average (1/8,071) (Deng et al., 2021). Notably, this study reported three unreported mutations in three genes (*ACADM*, *ACADS*, and *HLCS*) [c.893G>A (p.R298K), c.482G>A (p.S161N), and c.1544G>A (p.S515)] (Table 5) The three genes were derived from three diseases (SCADD, MCADD, and HCSD), and all of them were compound heterozygous mutations. These newly discovered mutations are convenient for providing further genetic counseling and enriching the database information of organic acid metabolism diseases. Most of the five diseases were inherited from parents. The results of this study suggest that although the incidence of organic acid metabolic diseases is low in this region, the proportion of people carrying the gene mutations for organic acid metabolic diseases is relatively high.

The combined FAOD incidence is 1/9,300 in reports from Australia, Germany, and the United States; however, it is rarely identified in Asian countries. In this study, the incidence of FAOD was 1/7,569. This was higher than that in Zhejiang Province (1/32,354) (Huang et al., 2011), Suzhou City (1/10,041) (Luo et al., 2018), Shanxi Province (1/9,015) (Dai et al., 2021) and the national average level of 1/10,917 (Lindner et al., 2010; Deng et al., 2021; Pereira et al., 2021). Furthermore, only one fatty acid metabolic disease was found in this region. The common gene mutations was *SLC22A5* c.1400C>G (p.S467C), with 90% of patients with PCD having this gene mutation, of which two patients maternally inherited PCD. Notably, the incidence of fatty acid metabolism in the Zhuzhou area was higher than that in other areas, and only one disease (PCD) was found, and its incidence is much higher than that of the Chinese population. This suggests a regionally high incidence of IMDs in this area.

Thus, by analyzing the screening and genetic diagnosis results of 90,829 newborns with IMDs in Zhuzhou, the disease spectrum, incidence, and clinical phenotype characteristics of amino acid metabolic, organic acid, and fatty acid oxidation disorders in newborns were determined. However, this study provides a different perspective. The incidence of fatty acid oxidation disorders in the Zhuzhou was relatively high, and the incidence of PCD was higher than that of other IMDs, suggesting a high regional incidence of fatty acid oxidation disorders in this area. The prevalence of organic acid metabolic diseases is high, and most patients with organic acid metabolism are inherited from their parents. Scientifically screening and diagnosing IMD the cases using biochemical methods and second-generation sequencing are required to achieve early screening and diagnosis and to provide strong support for reducing neonatal birth defects.

## 5 Conclusion

We have provided the most comprehensive mutation spectrum for IMDs. Additionally, we provided intensive genetic counseling and methods for early diagnosis and treatment. Our study may provide a baseline for genetic counseling, public education, and a reference for improving the prevention of IMDs.

## Data Availability

The original contributions presented in the study are included in the article/Supplementary Material, further inquiries can be directed to the corresponding author.
