# Supplementary material for: Prevalence of inherited metabolic disorders among newborns in Zhuzhou, a southern city in China
Source: Front Genet. 2024 Feb 6;15:1197151. doi: 10.3389/fgene.2024.1197151 (PMC10877023; doi:10.3389/fgene.2024.1197151)
Supplement: Supplementary file 1 [file DataSheet2.pdf]

Supplementary materilals 2

NGS Sequencing workflow information

Extraction of DNA: DBS or peripheral whole blood of suspected positive patients were referred to laboratory, and genomic DNA was extracted using the DNeasy Blood and Tissue Kit (QIAGEN Hilden, Germany)according to the manufacturer's protocol. DNA samples of the probands were quantified using a Qubit® dsDNA HS Assay Kit (Invitrogen, Carlsbad, CA, USA).

library preparation of multiplex： Genomic DNA was sheared to an approximate mean fragment length of 200-base pair (bp) using the Covaris LE220 (Covaris, Woburn, MA). Sheared DNA was used for library preparation of targeted regions by multiplex polymerase chain reaction (PCR). The library concentration and amplicon size were determined using an Agilent High Sensitivity DNA Kit(Agilent, Santa Clara, CA, USA).

Sequencing： The prepared sample libraries were sequenced by Illumina NextSeq 500 platform (Illumina Inc., San Diego, CA, USA) in paired end mode, generating 150-bp paired end reads.

Data Analysis： and the data were analyzed by NextSeq 500 Reporter.The variants were analyzed using an automatic false-positive filtering pipeline to ensure specificity and sensitivity when the allele frequency was ≥ 5%. ANNOVAR was performed against dbSNP (v138), 1000Genome, and ESP6500 to annotate the SNPs, insertions, and deletions. Only missense, stop-gain, frameshift, and non-frameshift indel mutations were retained for gene rearrangement analysis.
