# Supplementary material for: Prevalence of inherited metabolic disorders among newborns in Zhuzhou, a southern city in China
Source: Front Genet. 2024 Feb 6;15:1197151. doi: 10.3389/fgene.2024.1197151 (PMC10877023; doi:10.3389/fgene.2024.1197151)

Supplementary 3

some specific gene mutations of genetic metabolic diseases(PAHD、MET、MCADD、HCSD 、GA-I、PCD) are shown by sanger sequencing.

PAH c.728G>A(p.R243Q) c.1174T>A(p.F392I)

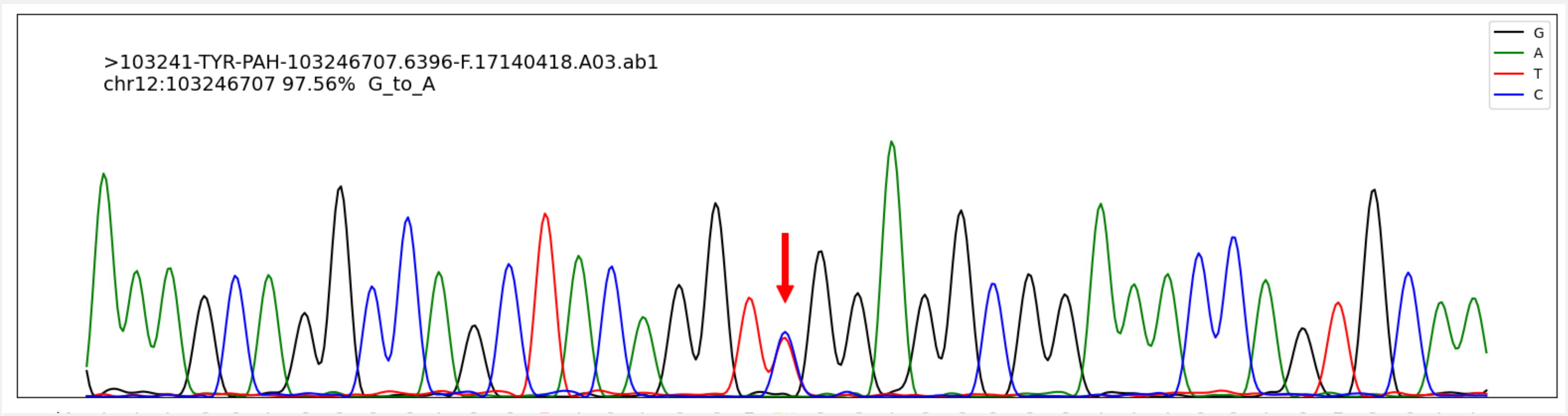

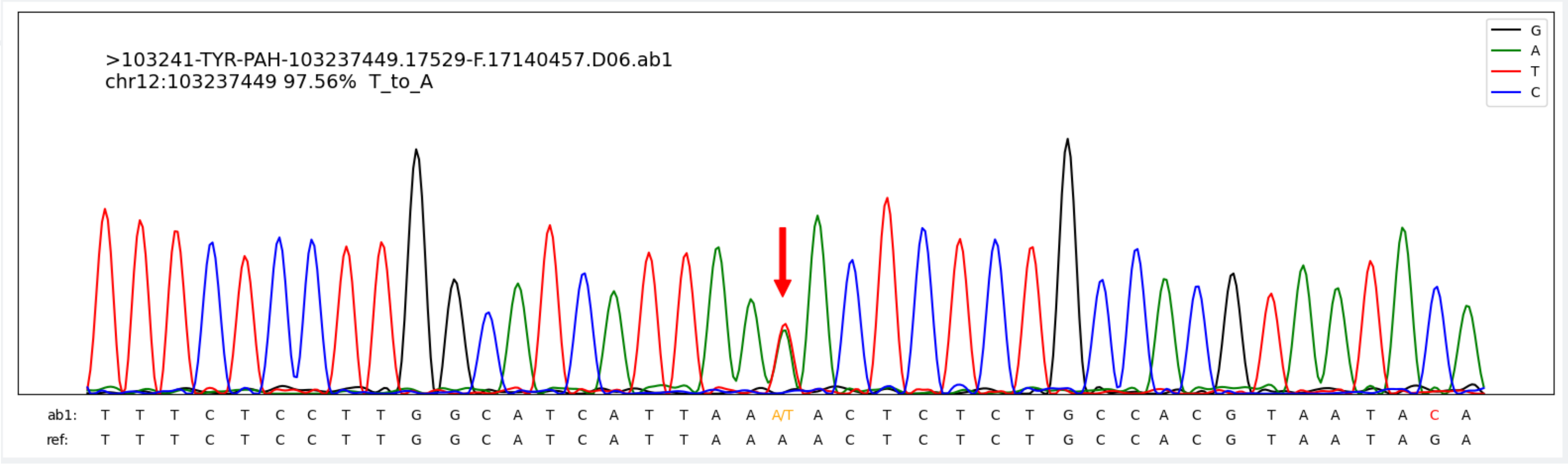

Hypermethioninemia(MET)

MAT1A c.433G>A(p.E145K) c.529C>T(p.R177W)

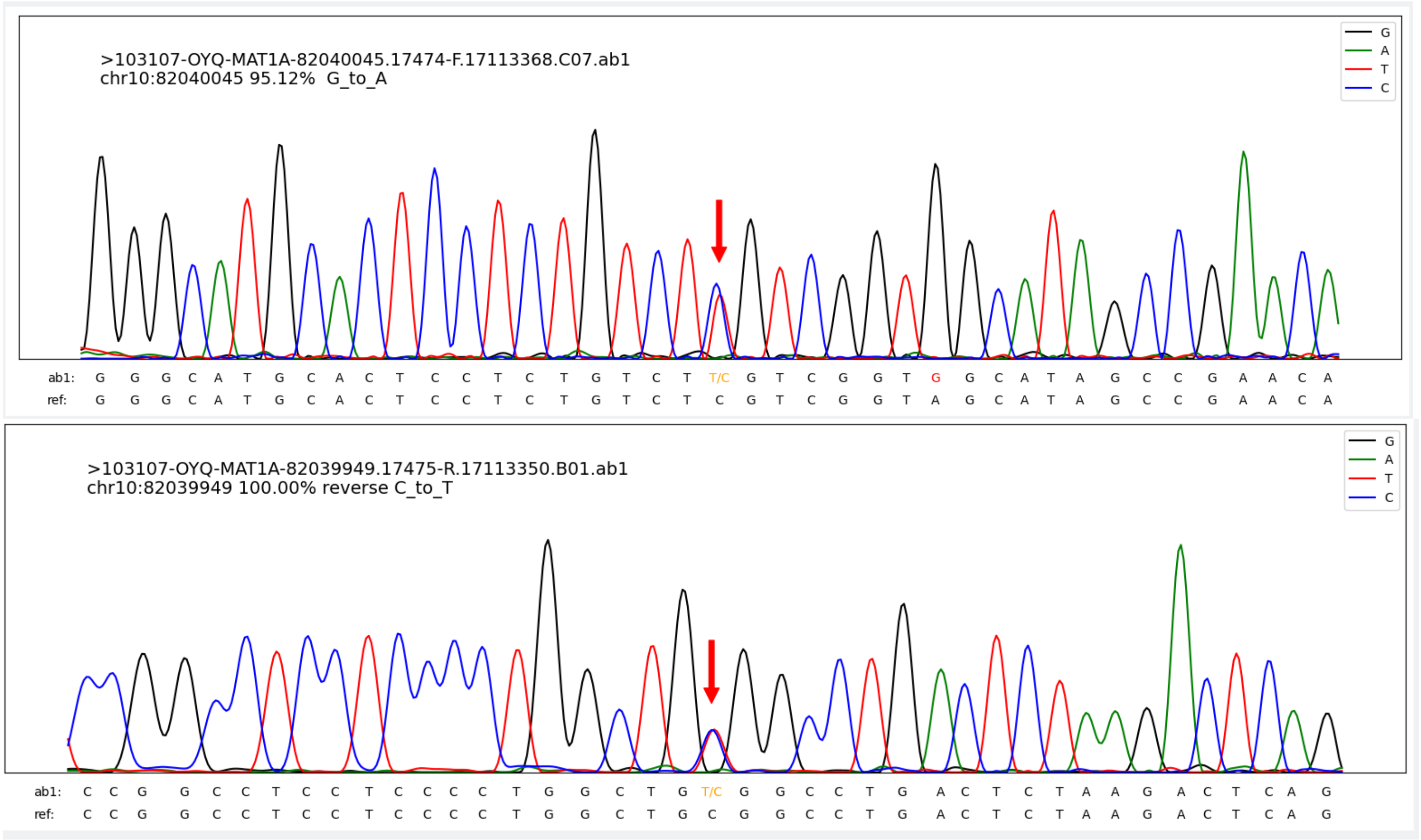

Middle/branched chain

acyl-CoA dehydrogenase deficiency (MCADD)

ACADS c.482G>A(p.S161N) c.1031A>G(p.E344G)

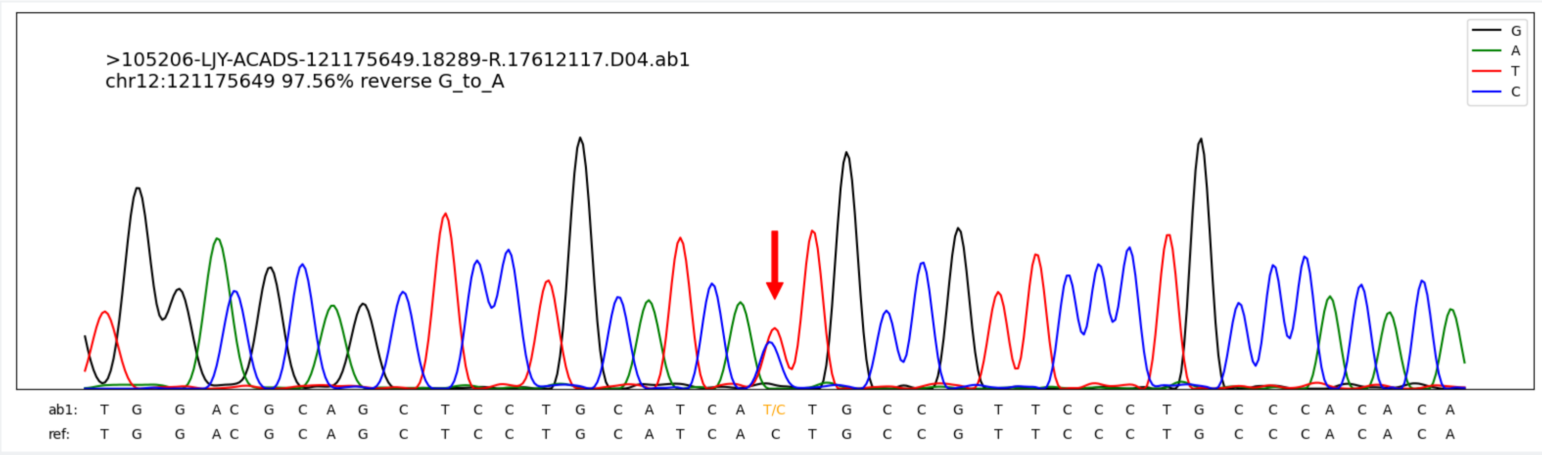

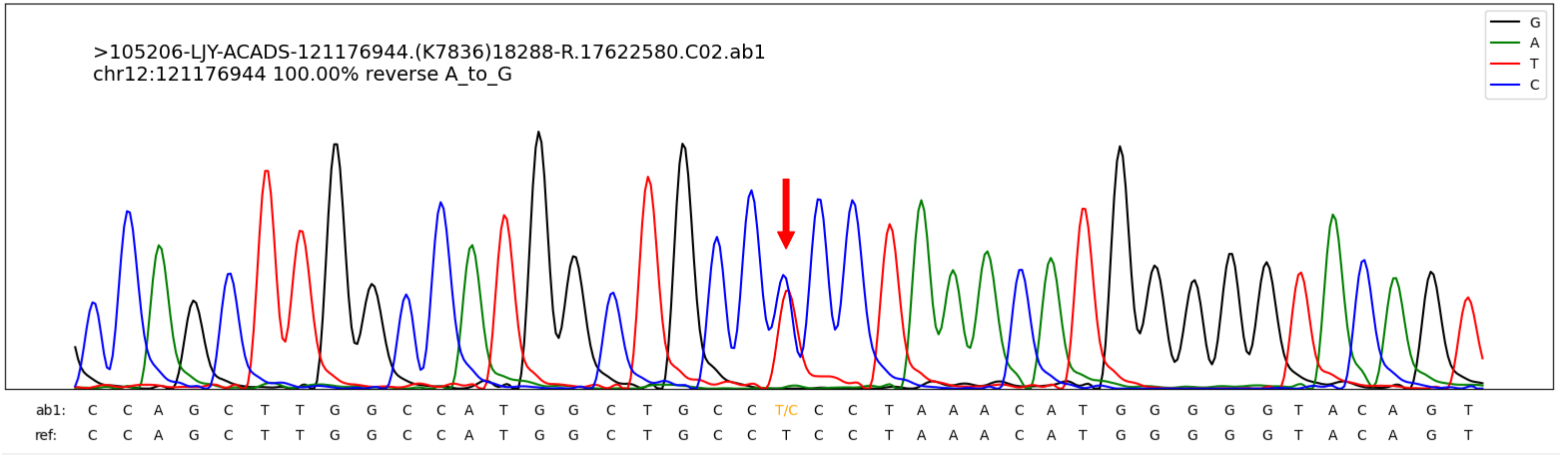

Holocarboxylase synthetase deficiency（HCSD)

HLCS　c.1544G>A(p.S515)　c.1522C>T(p.R508W)

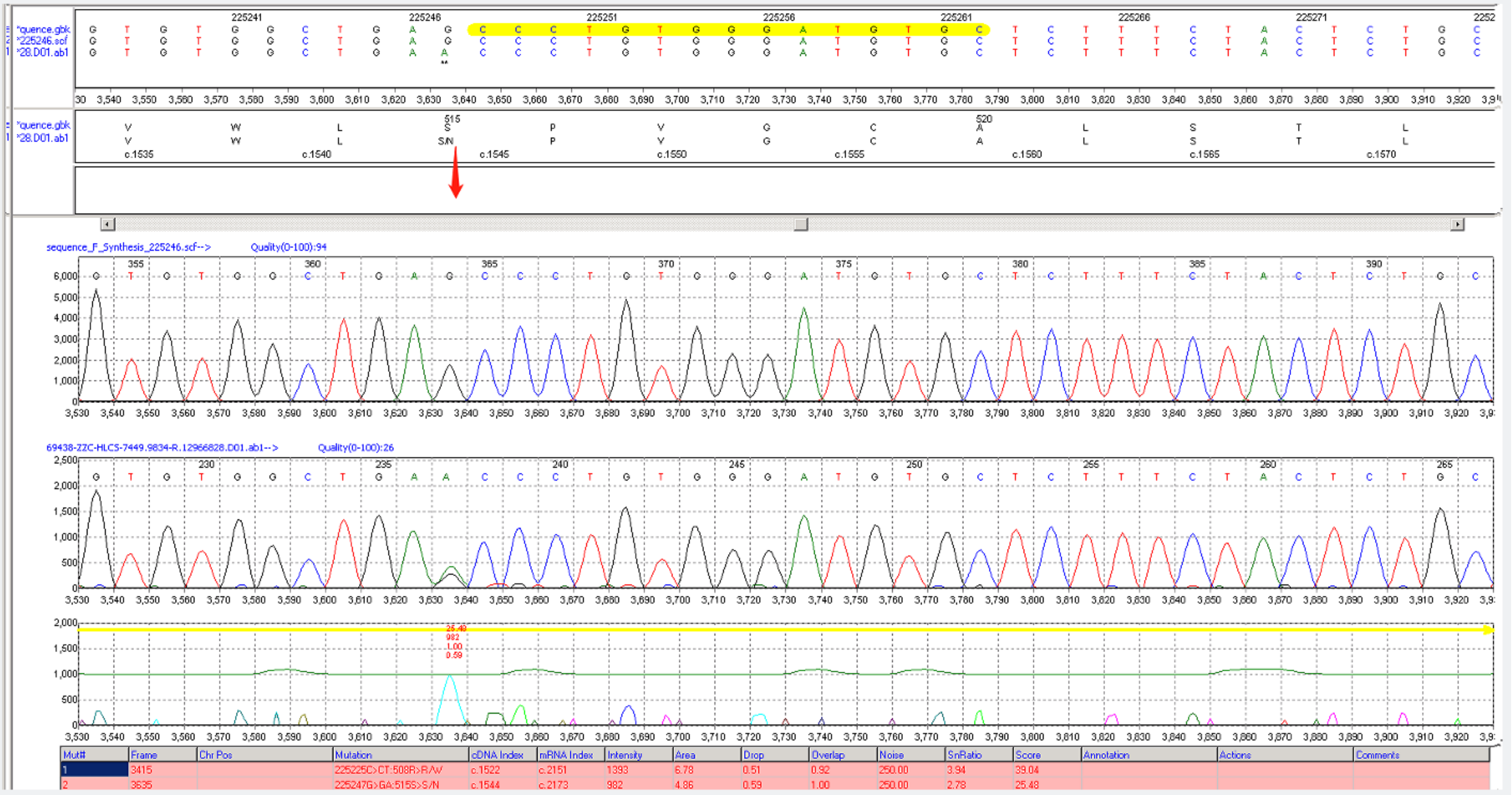

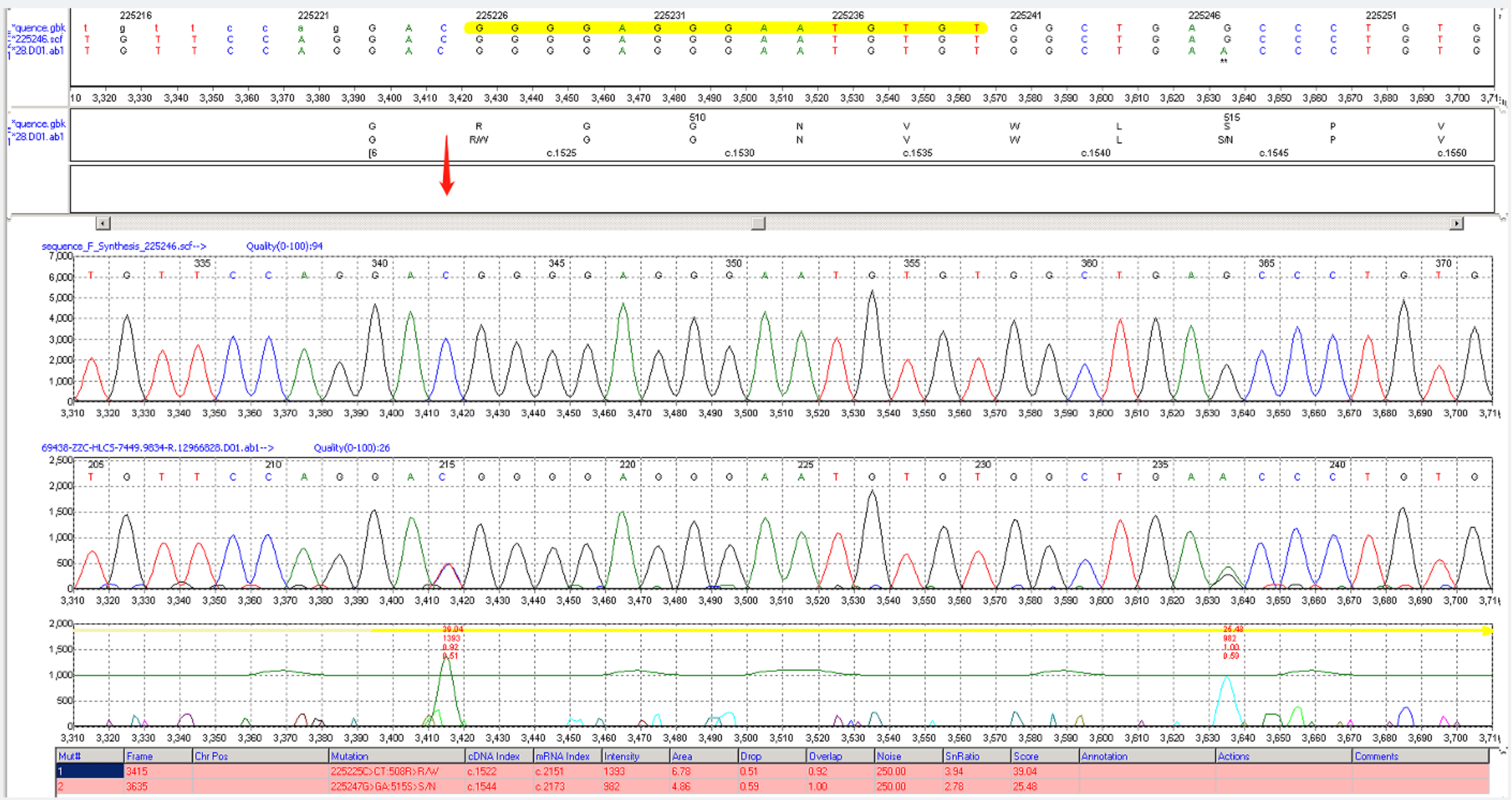
sequence_F_Synthesis_225246.scf-->
Quality(0-100):94
69438-ZZC-HLCS-7449.9834-R.12966828.D01.ab1-->
Quality(0-100):26
c.1525
c.1530
c.1535
c.1540
c.1545
c.1550
Mut#
Frame
Chr Pos
Mutation
cDNA Index
mRNA Index
Intensity
Area
Drop
Overlap
Noise
SnRatio
Score
Annotation
Actions
Comments
1
3415
225225C>CT:508R>R/W
c.1522
c.2151
1393
6.78
0.51
0.92
250.00
3.94
39.04
2
3635
225247G>GA:515S>S/N
c.1544
c.2173
982
4.86
0.59
1.00
250.00
2.78
25.48

Glutaric acidemia type I (GA-I)

GCDH c.416C>T(p.S139L) c.892G>A(p.A298T)

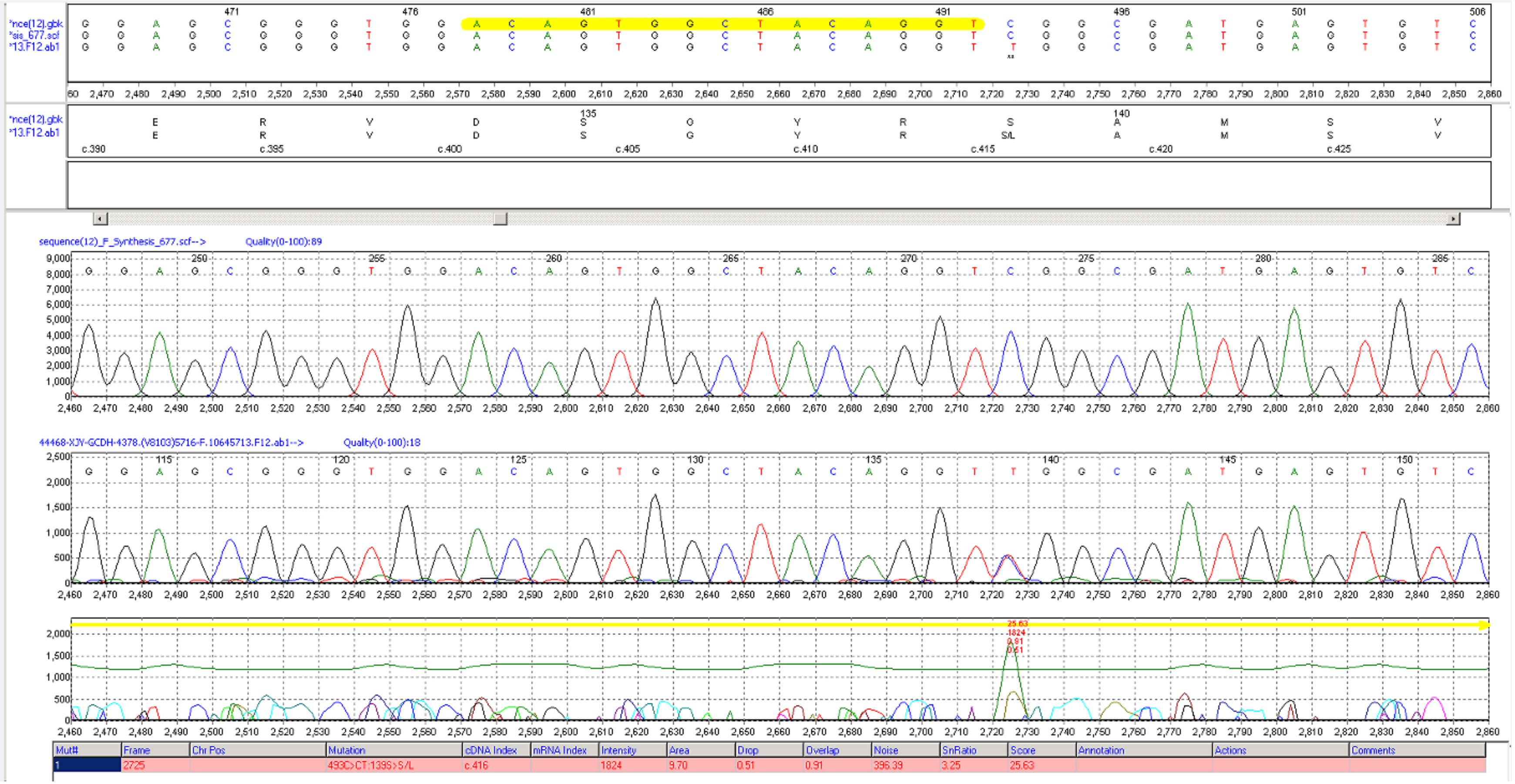

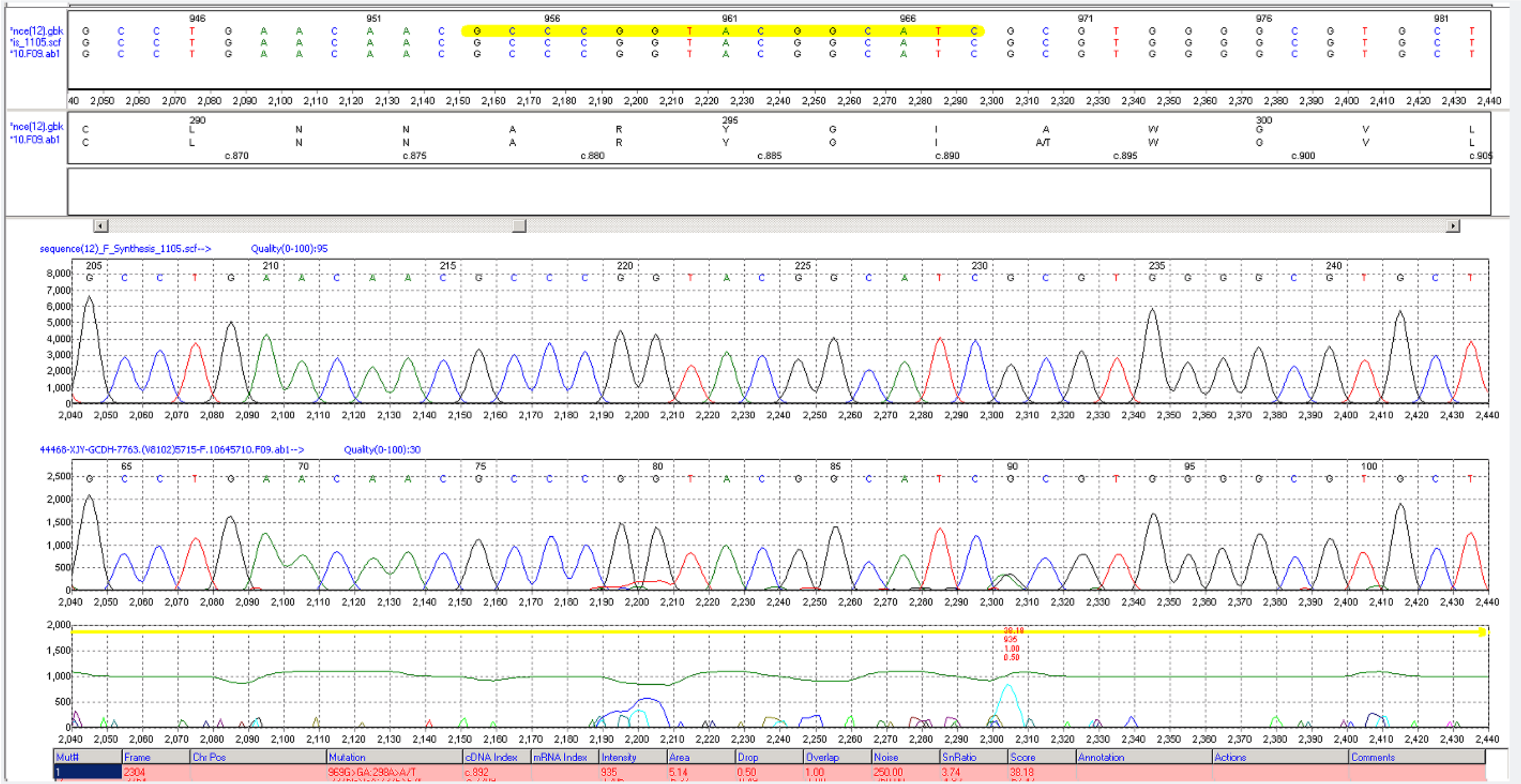
*nce(12).gbk
*is_1105.scf
*10.F09.ab1
sequence(12)_F_Synthesis_1105.scf-->
Quality(0-100):95
44468-XJY-GCDH-7763.(V8102)5715-F.10645710.F09.ab1-->
Quality(0-100):30
Mut# | Frame | Chr Pos | Mutation | cDNA Index | mRNA Index | Intensity | Area | Drop | Overlap | Noise | SnRatio | Score | Annotation | Actions | Comments
1 | 2304 | | 969G>GA:298A>A/T | c.892 | | 935 | 5.14 | 0.50 | 1.00 | 250.00 | 3.74 | 38.18

Primary carnitine deficiency (PCD)

SLC22A5　c.1400C>G(p.S467C)　c.1400C>G(p.S467C)

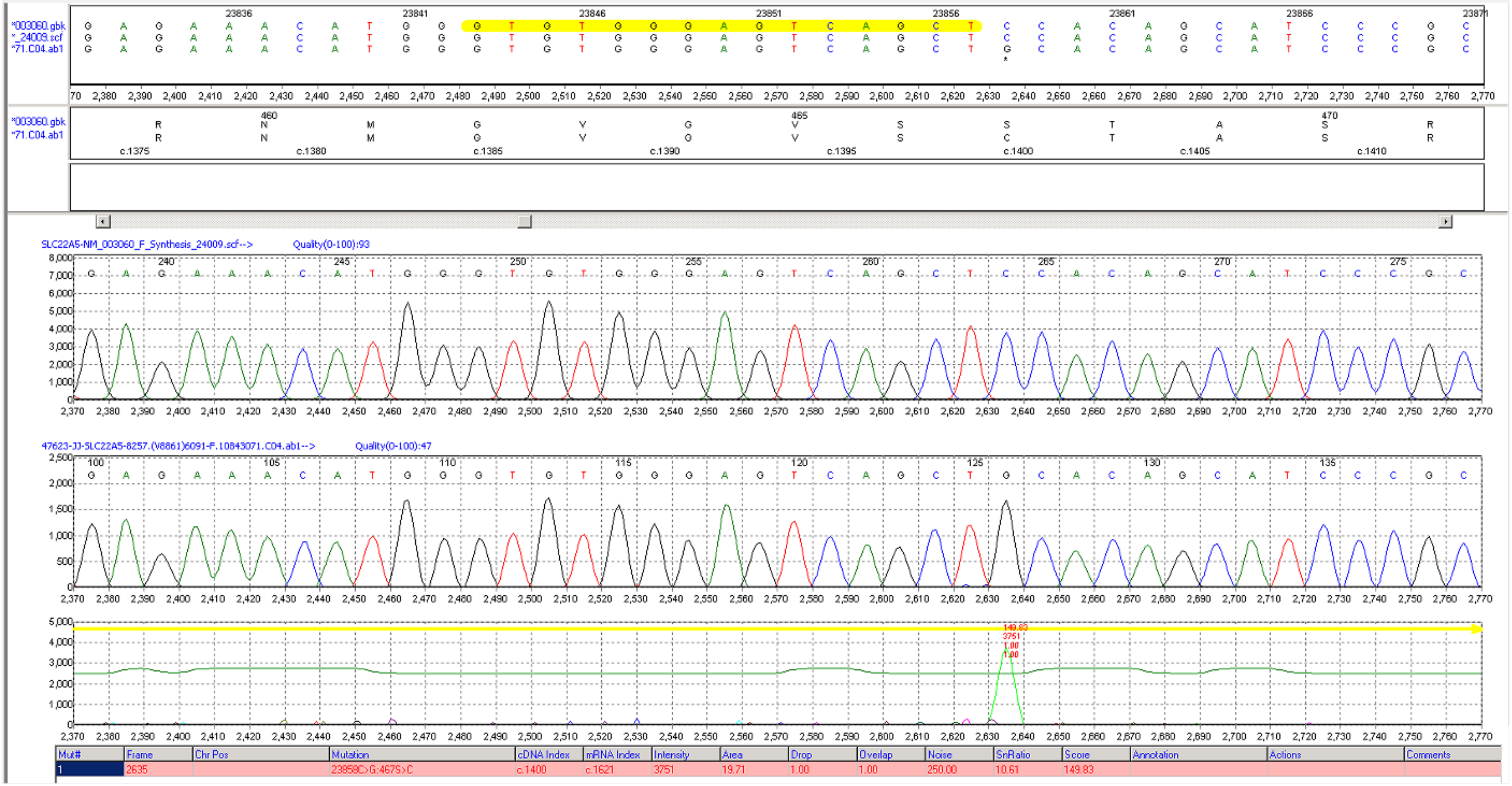

Supplement: Supplementary file 2 [file DataSheet3.pdf]
