# Supplementary material for: Prevalence of inherited metabolic disorders among newborns in Zhuzhou, a southern city in China
Source: Front Genet. 2024 Feb 6;15:1197151. doi: 10.3389/fgene.2024.1197151 (PMC10877023; doi:10.3389/fgene.2024.1197151)

Supplementary 1

Real mass spectra for 11 kinds of genetic metabolic diseases(PAHD、CD、HCY、MET、SCADD、MMA、MCADD、GA-I、PCD、OTCD) identified by mass spectrometry.

Phenylalanine hydroxylase deficiency (PAHD)

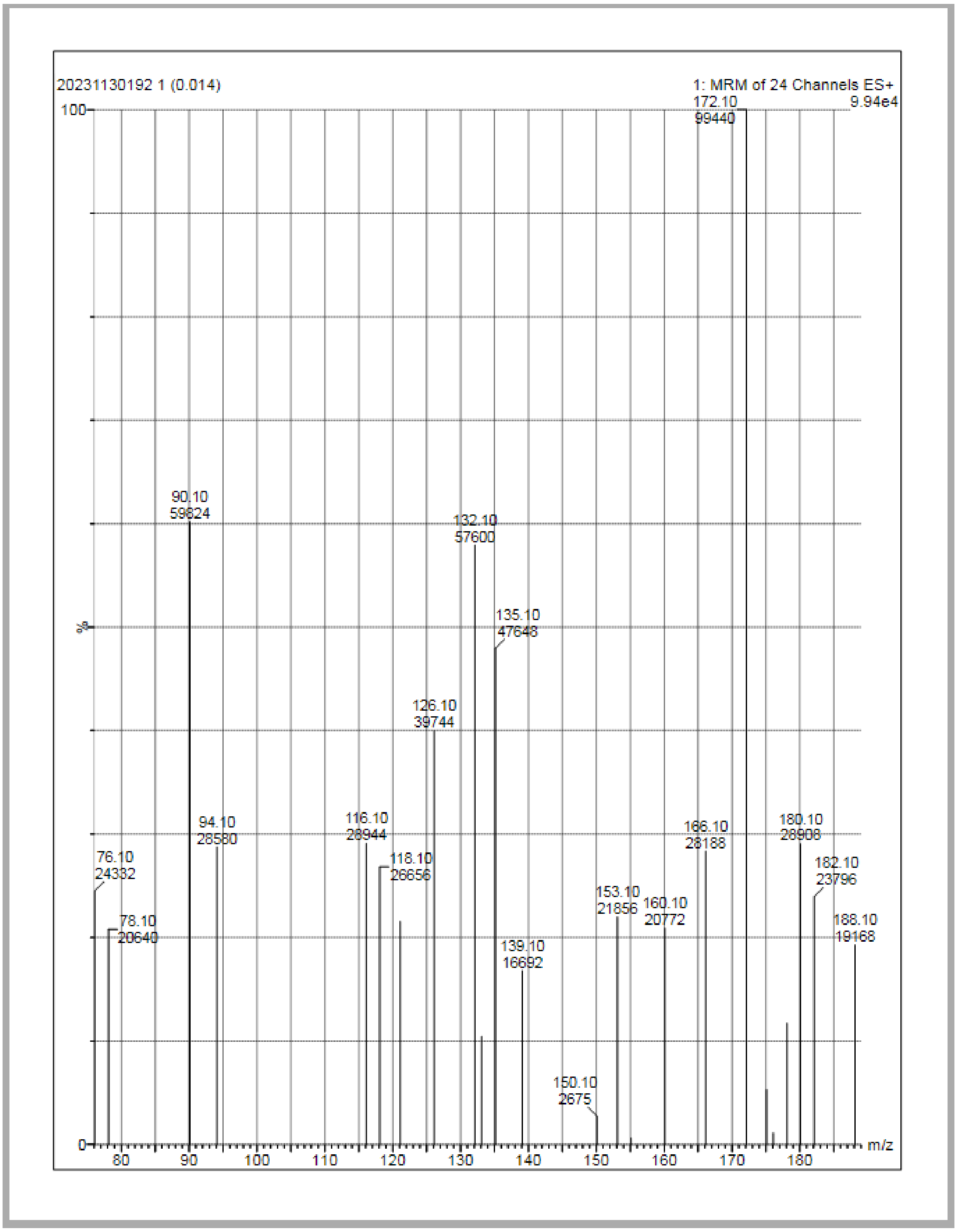

Citrin deficiency (CD)

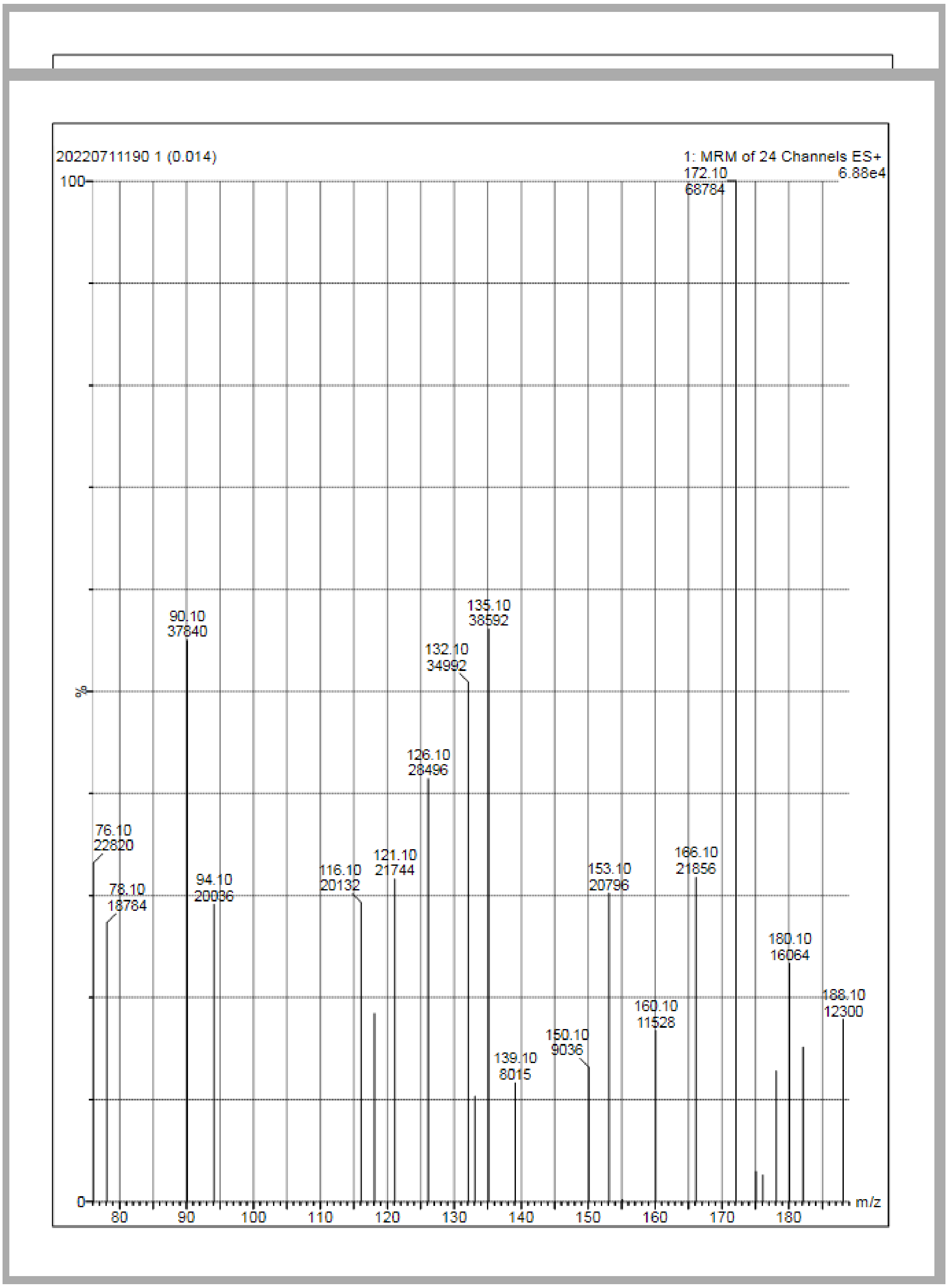

Homocysteinemia(HCY)

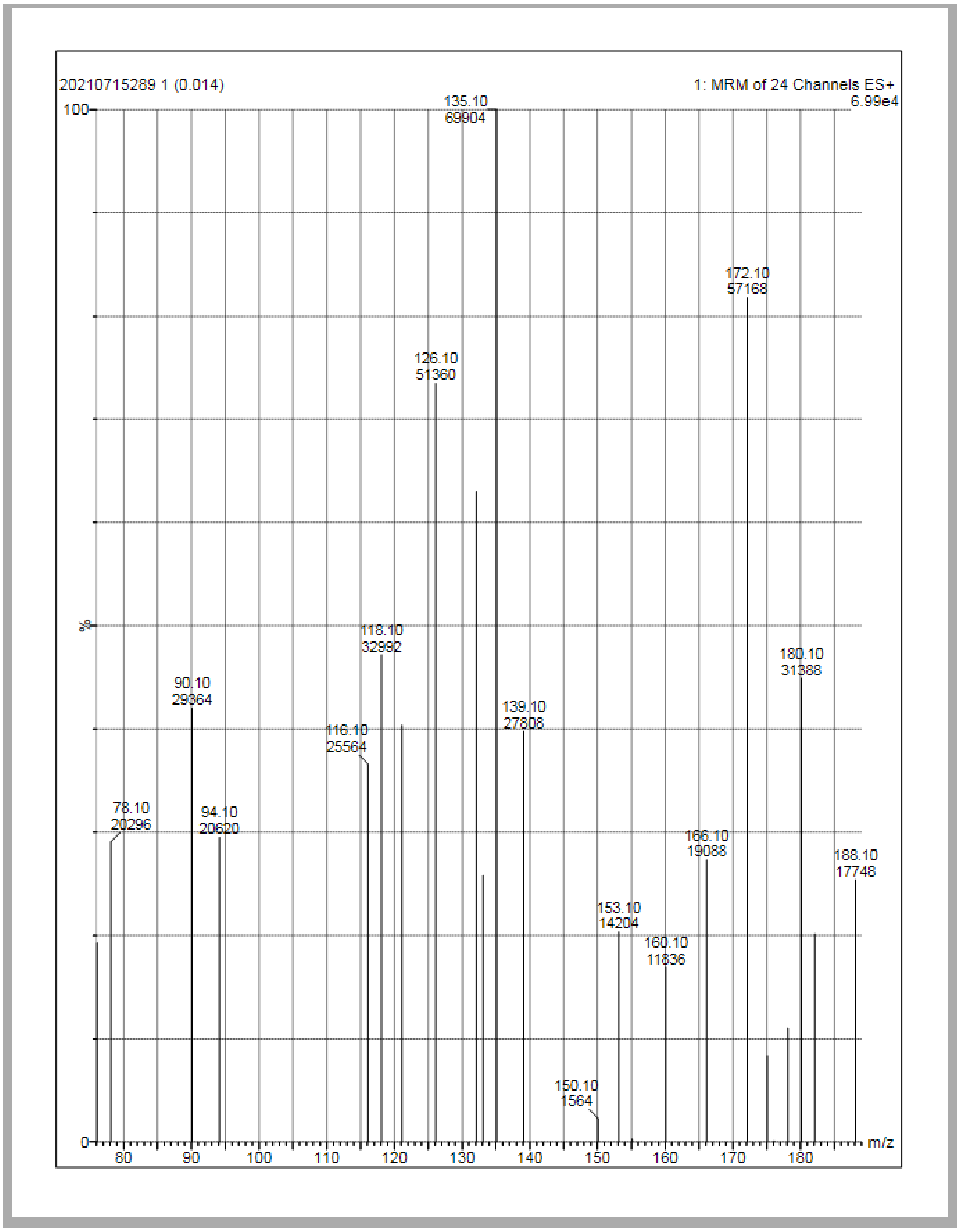

Hypermethioninemia(MET)

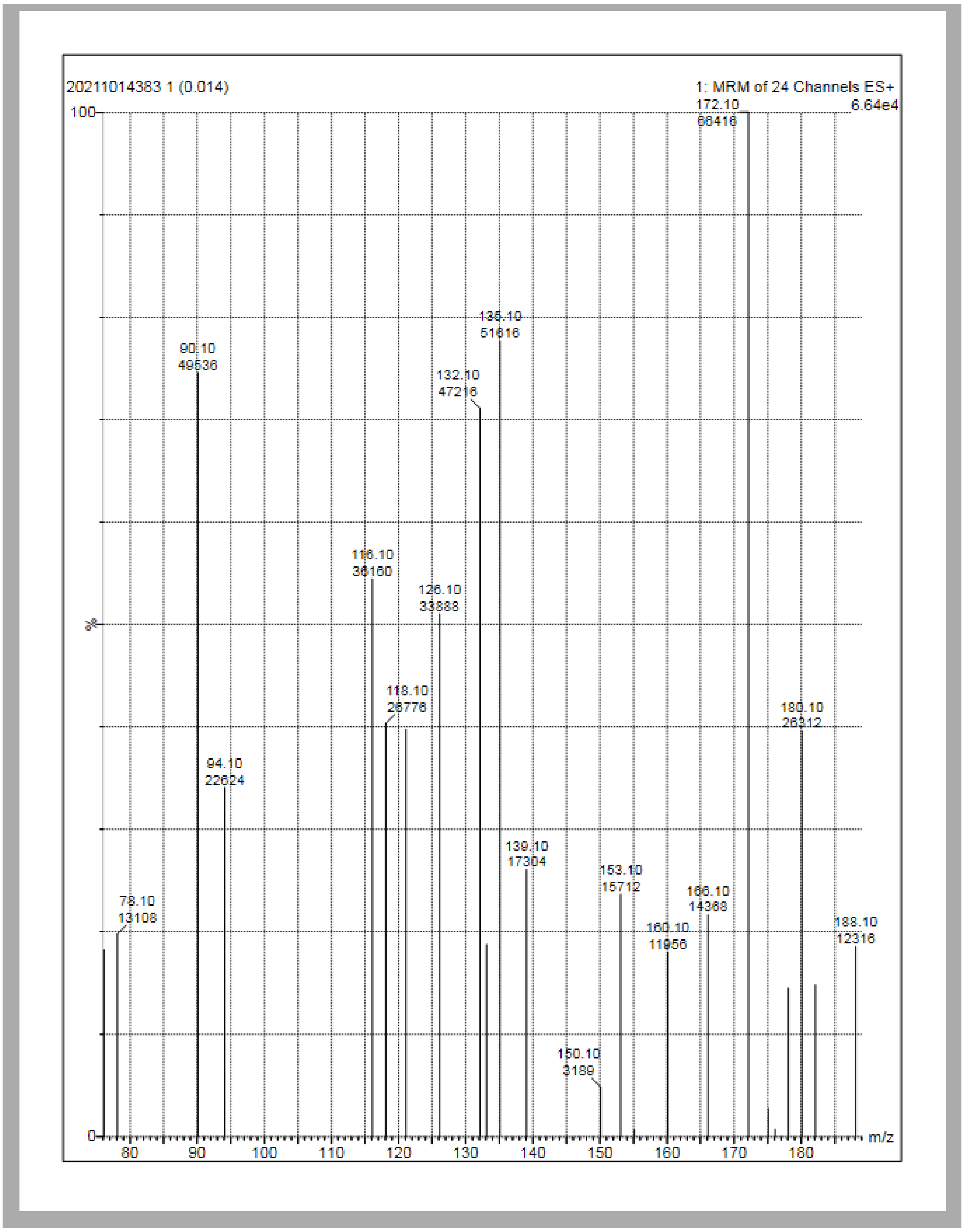

Short/branched chain acyl-CoA dehydrogenase deficiency (SCADD)

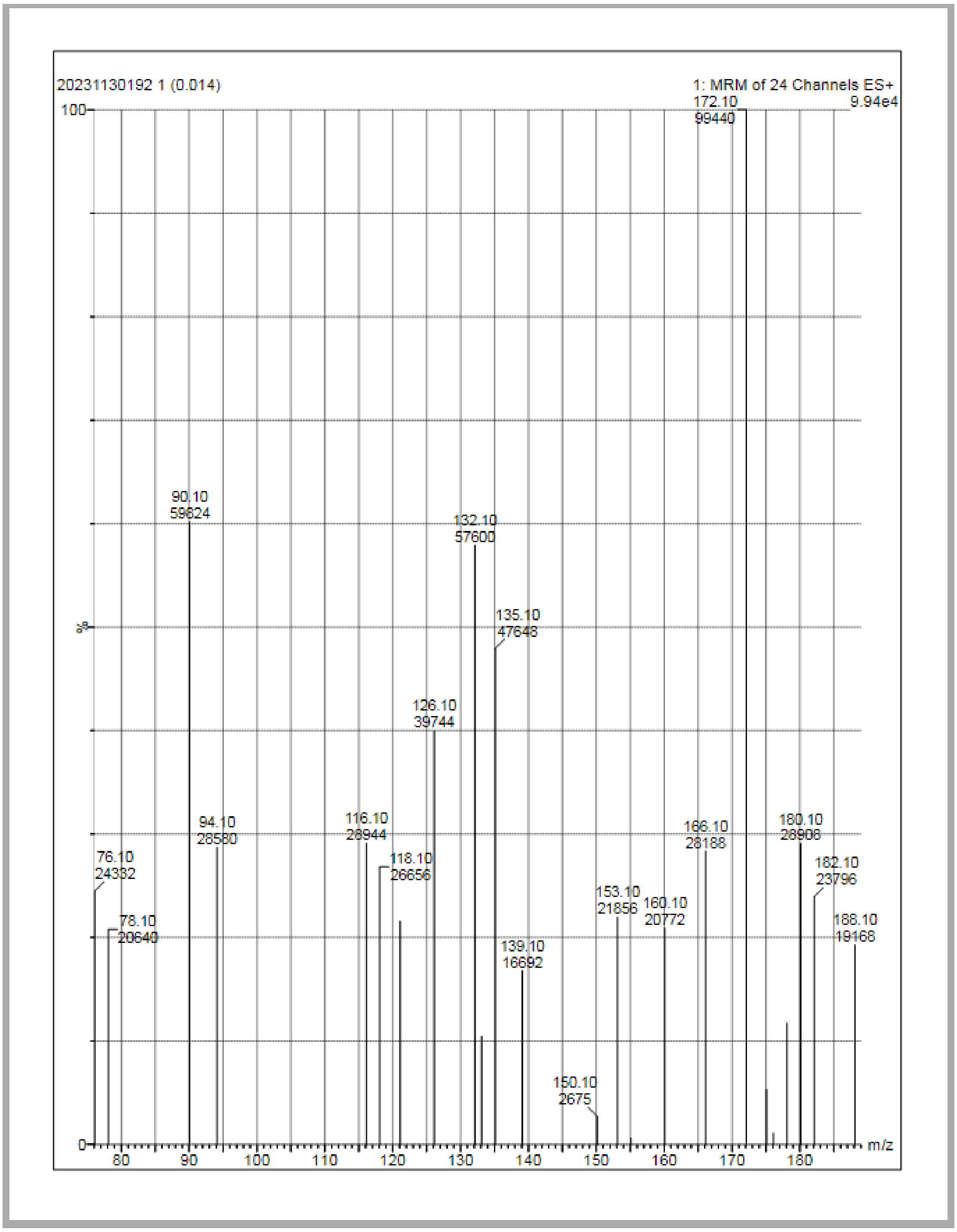

Methylmalonic academia (MMA)

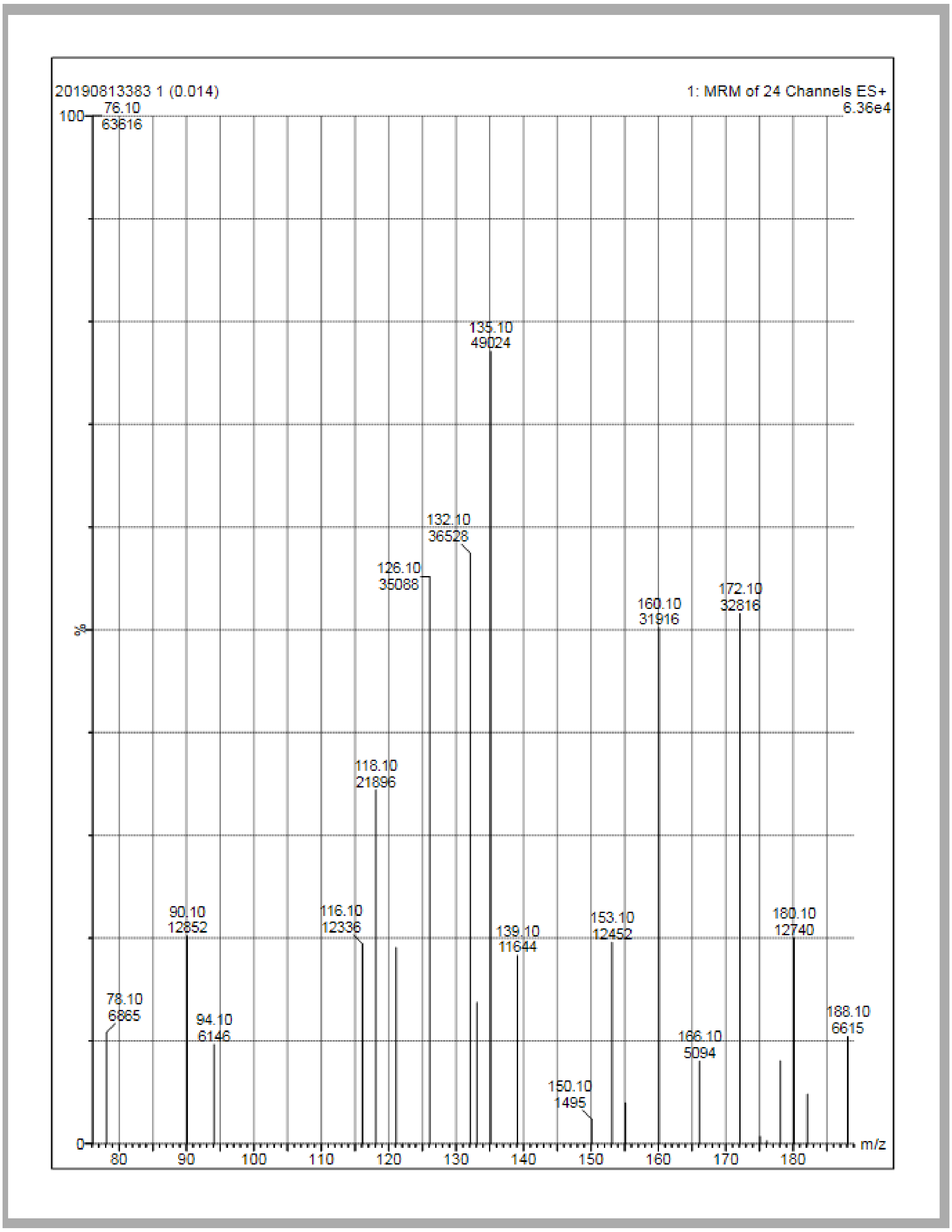

Middle/branched chain acyl-CoA dehydrogenase deficiency (MCADD)

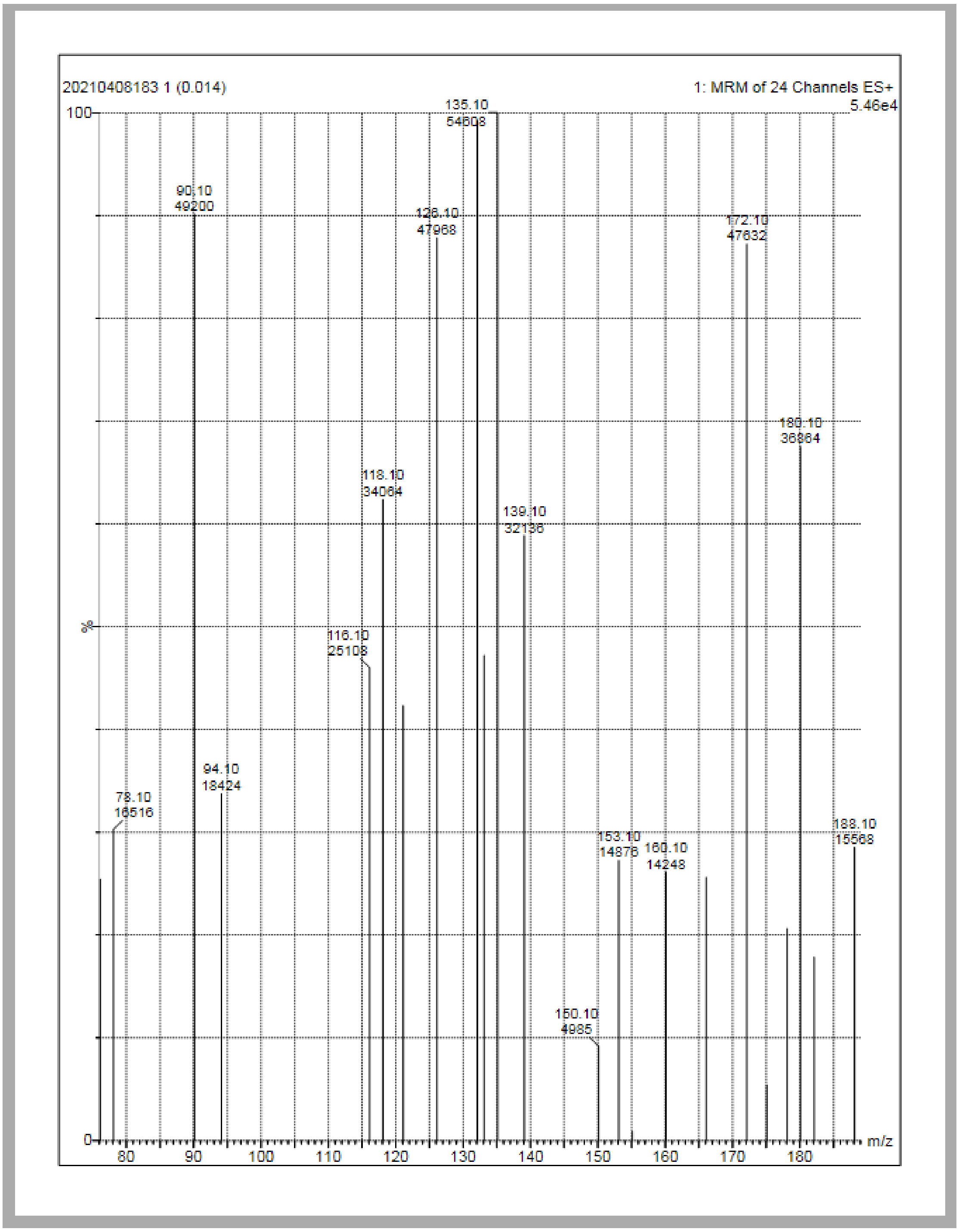

Holocarboxylase synthetase deficiency（HCSD)

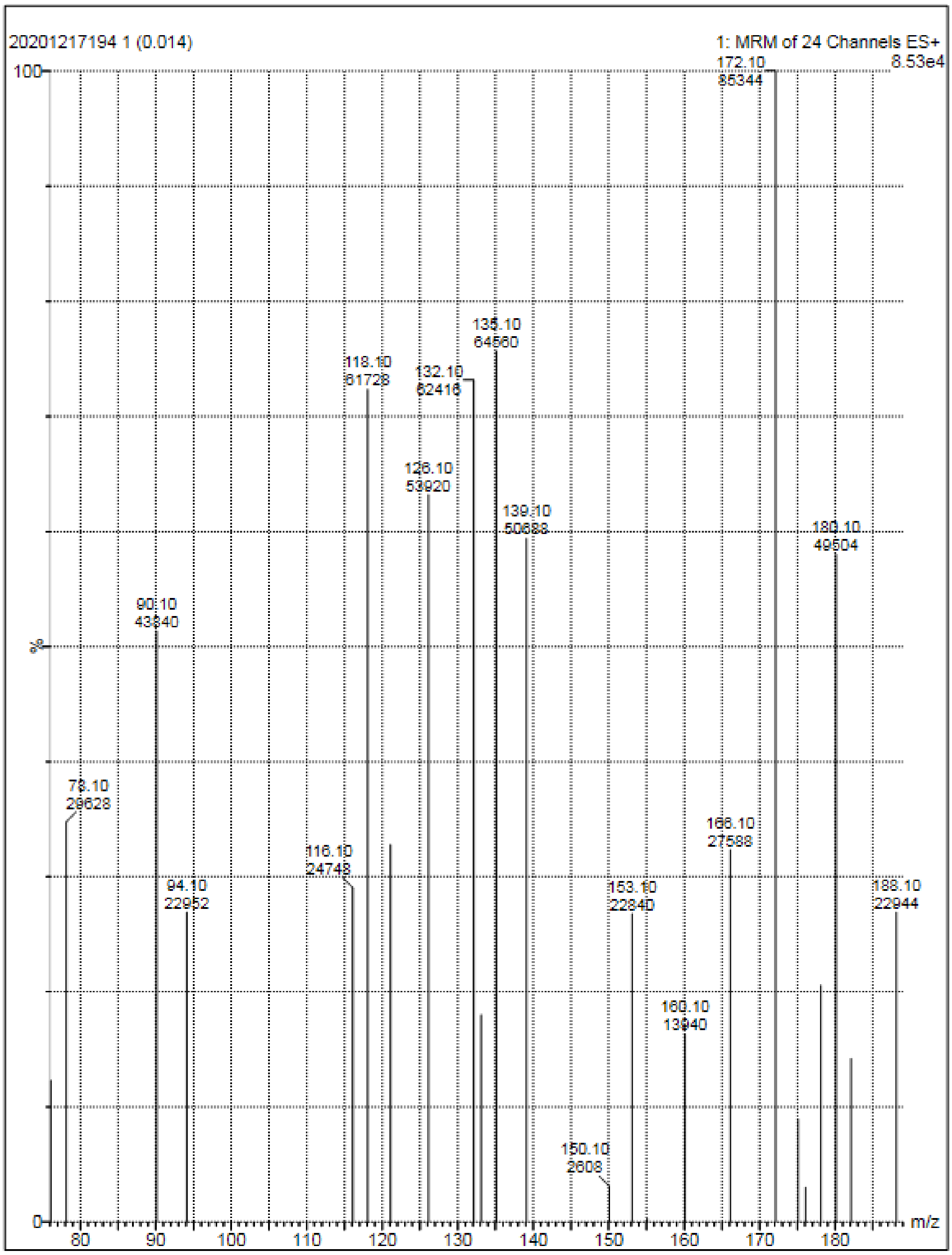

Glutaric acidemia type I (GA-I)

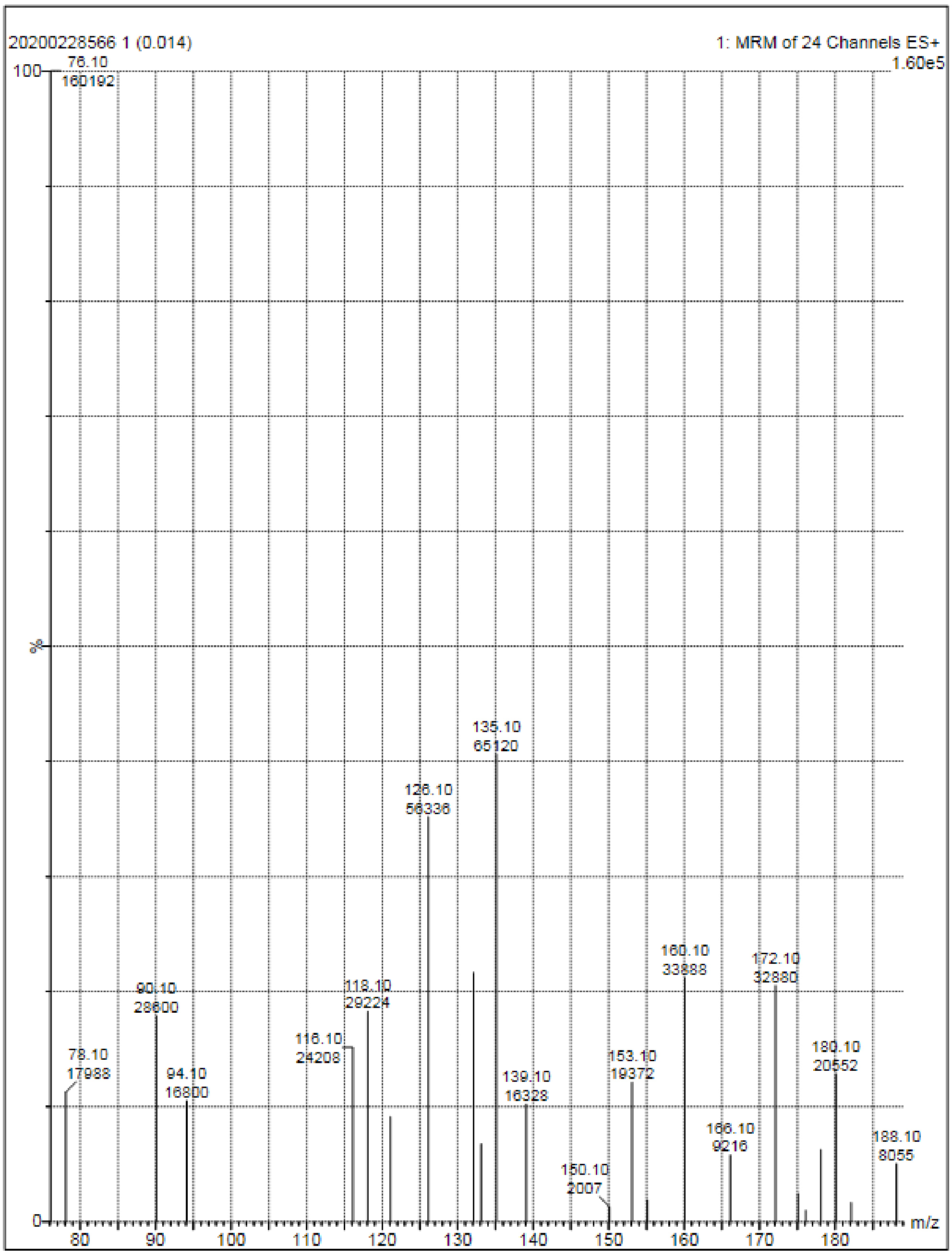

Primary carnitine deficiency (PCD)

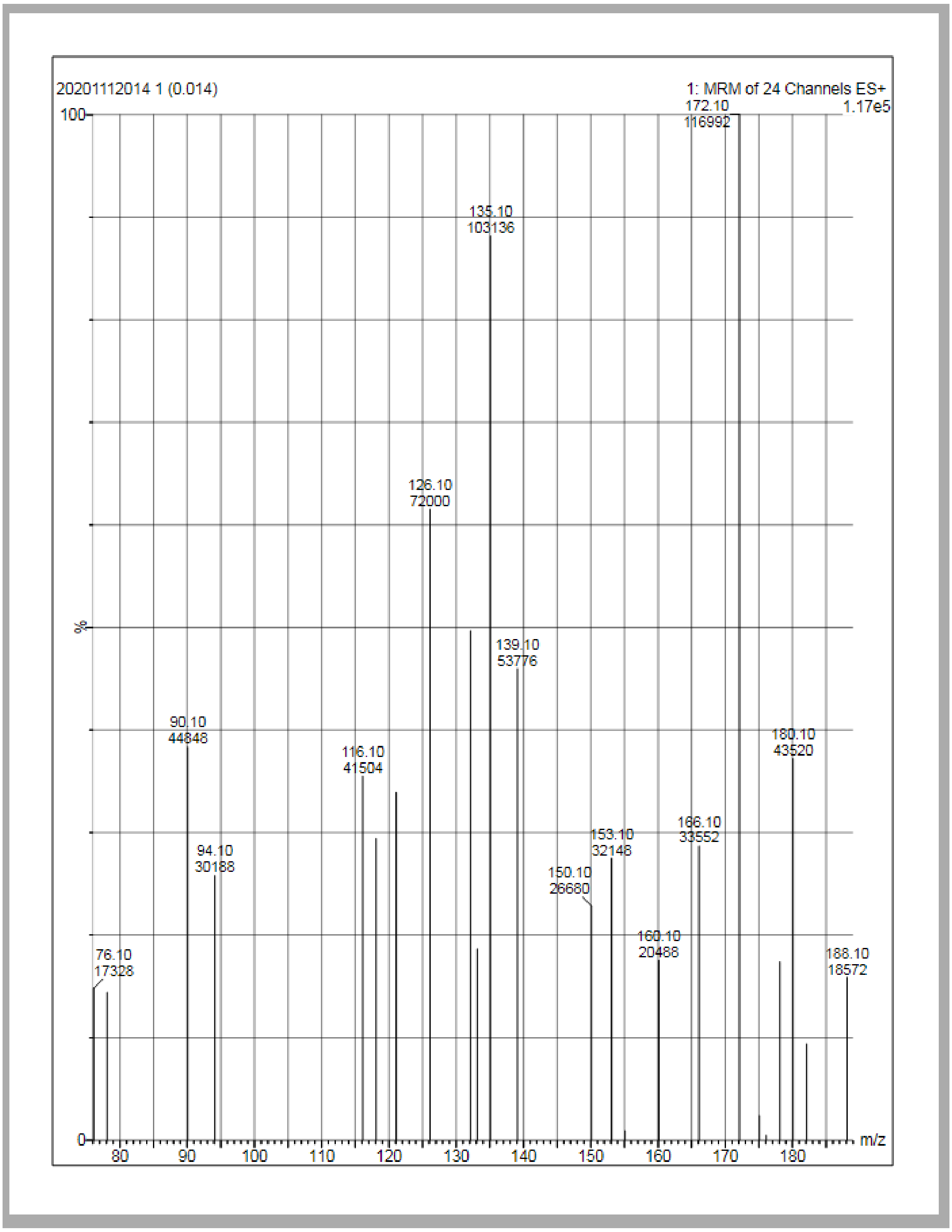

Ornithine transcarbamylase deficiency (OTCD)

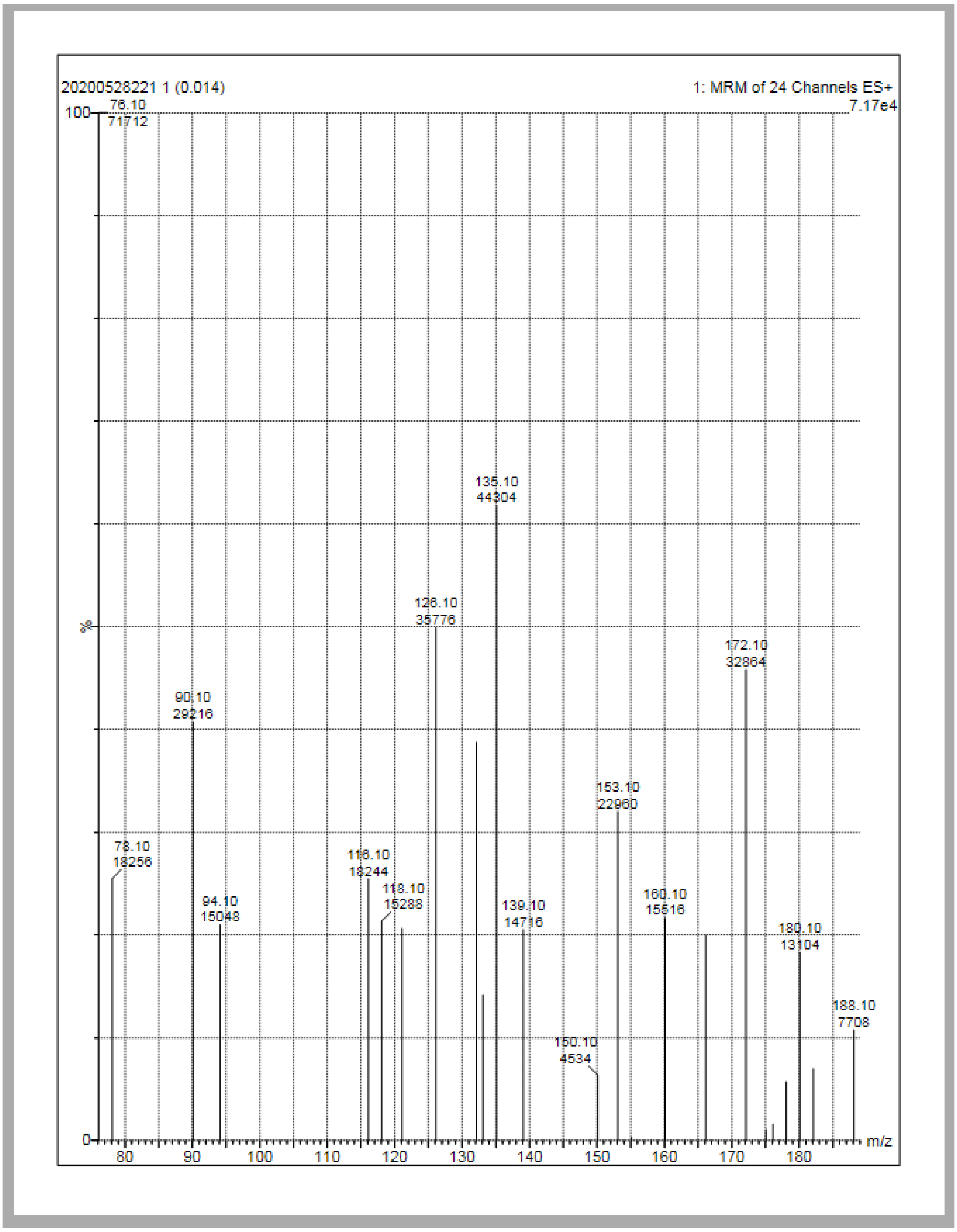

Supplement: Supplementary file 3 [file DataSheet1.pdf]
